# Treatment of Ulcers by Means of Opium

**Published:** 1850-05

**Authors:** F. C. Skey

**Affiliations:** Surgeon to St. Bartholomew’s Hospital


*Treatment of Ulcers by Means of Opium.*—By F. C. Skey, Esq., Surgeon to St. Bartholomew's Hospital.

In the summer of last year Mr. P., a gentleman residing in Montagu-square, a governor of St. Bartholomew's Hospital, placed himself under my charge, with a large chronic ulcer on the calf of the right leg. Its margin was irregular and jagged, and was elevated to at least one-third of an inch above the base of the ulcer, which base was pale and bloodless.—This wound was about the size of a man's extended hand, including the fingers, or even larger. It had existed for, I think, seventeen years. All remedies had failed, and the cure had long been abandoned as hopeless. I gave Mr. P. half a grain of opium night and morning. In three days he himself noticed the singular red color of the entire margin of the ulcer, which in a week extended over the base. I rolled and strapped the limb; the entire sore consequent on its depressed surface being placed beyond the influence of the pressure. Within two months that sore was reduced *to the size of half-a-crown*, and the *only remedy employed was half a grain of opium night and morning*, which, by-the-bye, had a very beneficial influence on his health.—*Med. Times*, *July* 21, 1846, *p.* 61.

